# *Clostridium difficile* clade 3 (RT023) have a modified cell surface and contain a large transposable island with novel cargo

**DOI:** 10.1038/s41598-019-51628-5

**Published:** 2019-10-25

**Authors:** Helen Alexandra Shaw, Ladan Khodadoost, Mark D. Preston, Jeroen Corver, Peter Mullany, Brendan W. Wren

**Affiliations:** 1 Department of Pathogen Molecular Biology, London School of Hygiene and Tropical Medicine, London, WC1E 7HT UK; 2 National Institute for Biological Standards and Controls (NIBSC), Blanche Lane, South Mimms, Potters Bar, Hertfordshire EN6 3QG UK; 3 Division of Microbial Diseases, University College London, 256 Gray’s Inn Road, London, WC1X 8LD UK; 4 Department of Medical Microbiology, Leiden University Medical Center, Leiden, the Netherlands

**Keywords:** Bacterial genetics, Bacteriology

## Abstract

The major global pathogen *Clostridium difficile* (recently renamed *Clostridioides difficile*) has large genetic diversity including multiple mobile genetic elements. In this study, whole genome sequencing of 86 strains from the poorly characterised clade 3, predominantly PCR ribotype (RT)023, of *C. difficile* revealed distinctive surface architecture characteristics and a large mobile genetic island. These strains have a unique sortase substrate phenotype compared with well-characterised strains of *C. difficile*, and loss of the phage protection protein CwpV. A large genetic insertion (023_CTnT) comprised of three smaller elements (023_CTn*1-3*) is present in 80/86 strains analysed in this study, with genes common among other bacterial strains in the gut microbiome. Novel cargo regions of 023_CTnT include genes encoding a sortase, putative sortase substrates, lantibiotic ABC transporters and a putative siderophore biosynthetic cluster. We demonstrate the excision of 023_CTnT and sub-elements 023_CTn*2* and 023_CTn*3* from the genome of RT023 reference strain CD305 and the transfer of 023_CTn*3* to a non-toxigenic *C. difficile* strain, which may have implications for the use of non-toxigenic *C. difficile* strains as live attenuated vaccines. Finally, we show that the genes within the island are expressed in a regulated manner in *C. difficile* RT023 strains conferring a distinct “niche adaptation”.

## Introduction

*C. difficile* is a nosocomial pathogen with at risk groups including the elderly and immunocompromised. However, infants are frequently asymptomatically colonised and represent a potential reservoir for pathogenic strains^1^. Recently, the reported incidence of *C. difficile* infection in the community has increased, which is often associated with younger patients and less severe infections^2^.

The cell surface of *C. difficile* is covered with a proteinaceous S-layer comprised mainly of SlpA, with other minor but important S-layer proteins in the cell wall protein (CWP) family^3^. These are non-covalently bound to the cell wall by interaction with the anionic cell wall polymer PSII^4^. Minor CWPs include Cwp66 putatively involved in adhesion and CwpV, a phase variable protein involved in cell-cell interaction and protection from phage^3,5^. In addition to S-layer proteins are sortase substrates, covalently anchored to the peptidoglycan cell wall, which in many Gram-positive bacteria have been implicated in pathogenesis and colonisation^6^. In *C. difficile* the sortase substrate CD2831 has been demonstrated to bind to collagen, suggesting a role in colonisation^7,8^.

The genome of *C. difficile* is highly variable, with core genes constituting only approximately a quarter (947–1033) of the predicted total coding sequence^9^. Core genes can be involved in horizontal gene transfer with the toxin genes proven to transfer and S-layer protein loci implied by genome analysis to have transferred between strains^10–12^. Additionally, in RT023 strains a large glycosylation locus has been observed within the S-layer cluster, and an additional transposable element within the toxin pathogenicity locus, PaLoc^11–14^. Mobile genetic elements including conjugative transposons, now more commonly referred to as ICE (integrative and conjugative elements), further diversify the genome content of *C. difficile* strains. ICE within the *C. difficile* genome are often related, with variations of the eight known mobile elements in the genome of reference strain 630 found in other strains of *C. difficile* with a consistent content of genetic cargo^15,16^. Acquisition of loci could be related to outbreaks, such as observed in an RT017 outbreak in a London hospital where strains harboured a transposon newly observed in *C. difficile* strains^17^. These occurrences add to the genome plasticity of the *C. difficile* species.

Clade 3, made up predominantly of RT023 strains, is the least characterised of the five known *C. difficile* clades and has strain CD305 as the assigned genome sequenced reference strain^14^. Here, we analyse the genomes of 86 clade 3 strains for alterations in their cell surface structure and demonstrate the presence of a large transposable element (023_CTnT), which may confer enhanced colonisation and survival in the human intestine.

## Results

### Clade 3 strains contain a truncated protease PPEP-1 resulting in permanent association of cell wall protein CD2831

**Figure 1:**
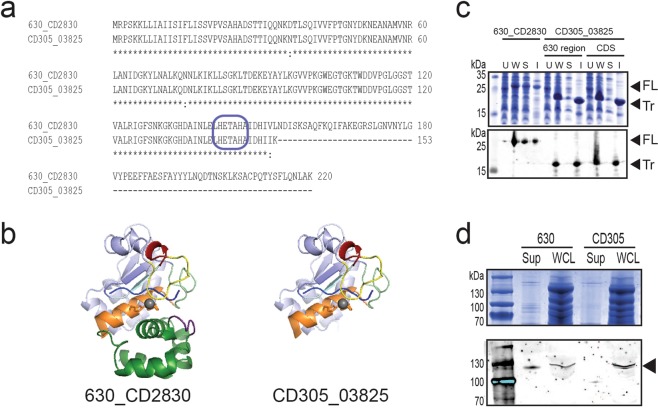
PPEP-1 is inactive in RT023 resulting in stable anchoring of sortase substrates to the cell wall. PPEP-1 from RT023 is insoluble in *E. coli* and inactive in *C. difficile*. (**a**) Translated protein sequence alignment of PPEP-1 in 630 and CD305 showing high sequence identity (*) until truncation of the CD305 protein after the putative active site (blue box). (**b**) Structural prediction of PPEP-1 in 630 and CD305. (**c**) Expression of 6xHisTag PPEP-1 from 630 and CD305 in *E. coli* by Coomassie staining and immunoblotting (Mouse anti-His 1:2,000, 680IRDye anti-mouse 1:2,000). U, uninduced; W, whole cell lysate; S, soluble; I, insoluble; FL, full length; Tr, truncated. Samples normalised to an OD 20/ml. (**d**) Localisation of sortase substrate CD2831 in *C. difficile* strains 630 and CD305 by Coomassie staining and immunoblotting (Mouse anti-CD2831 1:2,000, 680IRDye anti-mouse 1:2,000). Sup, supernatant; WCL, whole cell lysate. Black arrow indicates CD2831. Samples normalised to OD 50/ml. Full length gels are provided in Supplementary Fig. S1.

Sortase substrates are covalently anchored to the cell wall and are often involved in the colonisation and virulence of Gram-positive pathogens^6^. In *C. difficile*, through c-di-GMP regulation, the conserved protease PPEP-1 releases core genome sortase substrates CD2831 and CD3246 from the cell wall into the culture supernatant by cleavage of proline-rich motifs (recognition site: (V/I)NP|PVPP repeats), which has been suggested to provide regulated lifestyle switching^7,18–20^. There is a 2 bp deletion in the reference strain CD305 PPEP-1 homologue (CD305_03825) introducing an in frame stop codon (Fig. 1a) that was consistent between all strains in this clade (Supplementary Table S1). This deletion arises just after the characteristic HEXXH catalytic motif^19^, with structural prediction models showing a loss of the C-terminal loop (Fig. 1b). Sequence analysis of the substrate CD2831 homologue CD305_3823 showed a high sequence identity (95.2%), however substrate CD3246 homologue CD305_CD3434 has a 75 aa truncation at the C-terminus which removes five of the seven PPEP-1 cleavage sites (data not shown). Recombinant expression in *E. coli* showed CD305_03825 to form an insoluble truncated protein compared with PPEP-1 (630_CD2830) (Fig. 1c), suggesting misfolding and inactivation. A comparison of 630 and CD305 culture supernatants and whole cell lysates (WCLs) showed an absence of proteolytically released CD2831 in the supernatant of CD305 compared with 630 (Fig. 1d).

### Loss of CwpV in clade 3

**Figure 2:**
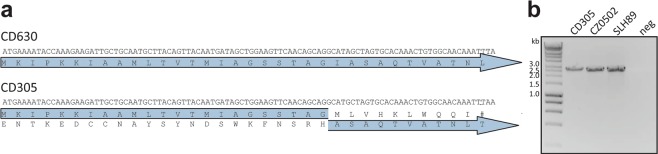
RT023 strains show an alteration of CwpV. CwpV contains an in frame stop codon in its signal sequence and has truncated repeats. (**a**) DNA sequence of first 102 bp of CwpV in 630 and CD305 genomes with adenosine deletion highlighted in red. Translated protein sequences represented in blue arrows with the frame shift represented as a break in CD305, # indicating a stop codon. The signal peptide cleavage site is indicated with a white arrow. (**b**) PCR of entire CwpV region in three strains of RT023 demonstrating the uniform length of CwpV representing two Type III repeats.

CwpV is a well characterised phase-variable S-layer protein with five known antigenically distinct “types” and is involved in protection against phage through prevention of phage DNA replication rather than through phage adsorption^5,21^. Analysis of the CD305 reference genome showed the presence of CwpV with just two Type III repeats. Furthermore, analysis of the gene sequence showed that a single base pair deletion had occurred within the signal peptide of CwpV, rendering a frame shift which leaves CwpV without a signal peptide (Fig. 2a). A PCR flanking *cwpV* was conducted on genomic DNA of clade 3 strains from patients in the UK, Europe and an animal source to confirm the truncation of this gene was not an error of WGS (Fig. 2b). This, along with Sanger sequencing of the product, confirmed the truncation to only two repeats for CwpV as well as the frame shift within the signal peptide, which was conserved in all 86 RT 023 strains analysed (Supplementary Table S1).

### RT023 strains contain a large genomic island insertion of three putative transposable elements

**Figure 3:**
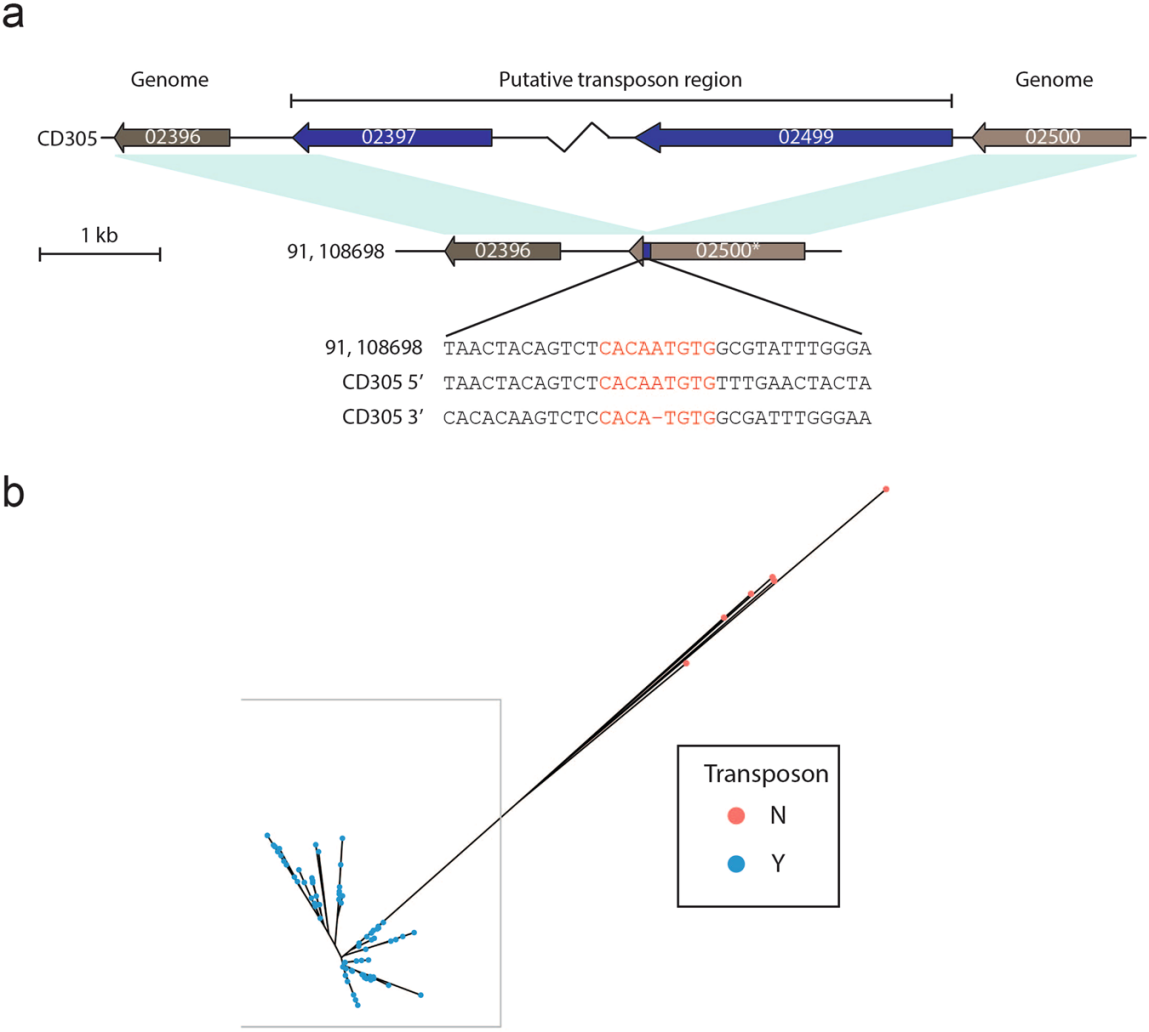
RT023 strains can contain a large novel genomic insertion at the 630_CTn*2* site. Analysis of genomic insertions shows a large transposable region in most strains of clade 3. (**a**) Schematic demonstrating the insertion site in strain CD305 and the empty site within strains 91 and 108698. Grey genes 02396 and 02500 are found within the core genome, with blue genes 02397 and 02499 representing the 5′ and 3′ termini of 023_CTnT. Sequence analysis of the empty site and 5′/3′ sequence of CD305 are shown. (**b**) Phylogenetic tree demonstrating the clustering of clade 3 strains from this study coloured according to presence (blue) and absence (red) of the transposon region.

Analysis of the CD305 genome reveals a 136.4 kb insertion within the region homologous to the 630_CTn*2* locus encompassing 103 predicted coding sequences (CD305_02397-02499) (Table 1, Fig. 3a), hereafter referred to as 023_CTnT. Downstream gene CD305_02396 and upstream gene CD305_02500 show homology to 630_CTn*2* insertion site flanking genes 630_CD0438 and 630_CD0406, respectively. Of the other 85 strains in our study, 79 (92.9%) contain 023_CTnT (Fig. 3b). Genomic analysis of strains 91, 108698, WCHCD103, WCHCD106 and WCHCD133^22^ and OX2183^11^ demonstrates they have an empty site, with CD305_02396 followed by CD305_02500 (Fig. 3). The empty site is occupied by an imperfect palindrome CACAATGTG, matching the sequence at the 5′ terminus of the CD305 putative transposon within CD305_02397 and the 3′ terminus within CD305_02500, the latter of which contains the perfect palindrome CACATGTG (Fig. 3a). When a phylogeny of clade 3 strains is constructed based on SNPs these six strains are all outliers from the core phylogeny of RT023 strains (Fig. 3b). There are 1578 SNPs across the entire region (96 non-coding, 410 non-synonymous and 1072 synonymous) with the majority clustering between CD305_0269 and CD305_02499.

**Table 1:** Putative transposable element insertion into clade 3 strains.

CD305 Locus Tag	Product	CD305 Locus Tag	Product	CD305 Locus Tag	Product
CD305_02397	Serine recombinase	CD305_02439	Site-specific serine recombinase, resolvase family	CD305_02469	Serine recombinase
CD305_02398	Conjugal transfer protein	CD305_02440	hypothetical protein	CD305_02470	conjugal transfer protein/hypothetical protein
CD305_02399	Transcriptional regulator	CD305_02441	type II toxin-antitoxin system PemK/MazF, mRNA interferase EndoA	CD305_02471	Helix-turn-helix protein
CD305_02400	DNA-directed RNA polymerase sigma-70 factor	CD305_02442	RNA polymerase sigma-70 factor, ECF subfamily	CD305_02472	RNA polymerase sigma-70 factor
CD305_02401	ABC transporter ATP-binding protein	CD305_02443	hypothetical protein	CD305_02473	signal transduction histidine kinase
CD305_02402	ABC transporter ATP-binding protein	CD305_02444	hypothetical protein	CD305_02474	Transcriptional regulatory protein SpaR/DNA-binding response regulator
CD305_02403	AraC family transcriptional regulator	CD305_02445	ylaC, RNA polymerase sigma factor	CD305_02475	hypothetical protein/nsuI protein
CD305_02404	ABC transporter ATP-binding protein	CD305_02446	hypothetical protein	CD305_02476	lantibiotic protection ABC transporter permease subunit, MutG family protein
CD305_02405	cobalt transporter, Ecf (energy-coupling factor)	CD305_02447	hypothetical protein/ABC transporter permease	CD305_02477	lantibiotic protection ABC transporter permease subunit, MutE/EpiE family protein
CD305_02406	membrane protein	CD305_02448	ABC transporter ATP binding protein	CD305_02478	lantibiotic protection ABC transporter, ATP binding protein srtF
CD305_02407	Thiazolinyl imide reductase	CD305_02449	HTH DNA binding protein, XRE family	CD305_02479	HTH DNA binding protein, XRE family
CD305_02408	Saccharopine dehydrogenase	CD305_02450	transglycosylase/CHAP domain protein	CD305_02480	glutamine amidotransferase, DJ-1/Pfp1 family protein, YdeA
CD305_02409	non-ribosomal peptide synthetase/pyochelin synthetase F	CD305_02451	srtB	CD305_02481	transcriptional regulator, deoR-like HTH DNA binding protein, YafY family transcriptional regulator
CD305_02410	non-ribosomal peptide synthetase	CD305_02452	hypothetical protein	CD305_02482	hypothetical protein/conjugative transposon protein
CD305_02411	2,3-dihydrozybenzoate-AMP ligase	CD305_02453	hypothetical protein/TraE family protein/TrsE protein	CD305_02483	membrane hypothetical protein,
CD305_02412	4′-phosphopantetheinyl transferase	CD305_02454	PrgI superfamily	CD305_02484	transcriptional regulator, AbrB family domain protein
CD305_02413	thioesterase	CD305_02455	hypothetical protein	CD305_02485	Conjugative transposon protein
CD305_02414	3-deoxy-7-phosphoheptulonate synthase	CD305_02456	TraG/TraD family protein/TsrK family protein conjugal transfer protein	CD305_02486	lysozyme like superfamily, peptidase NLPC_P60 superfamily
CD305_02415	Salicylate synthase	CD305_02457	hypothetical protein/ltrC-like protein	CD305_02487	MFS transporter, transposon protein
CD305_02416	transcriptional regulator (DtxR/MntR Manganese regulation)	CD305_02458	hypothetical protein/PcfB family protein	CD305_02488	AAA-like protein, ATP/GTP binding protein
CD305_02417	CDGSH-type zinc finger/transposase	CD305_02459	relaxase	CD305_02489	ArdA, antirestriction family protein, Tn916 like
CD305_02418	DEAD/DEAH box helicase/Type 1 restriction endonuclease subunit R	CD305_02460	topoisomerase/ltrC-like protein	CD305_02490	ArdA, antirestriction family protein, Tn916 like
CD305_02419	Hypothetical protein/Putative nucleotide binding	CD305_02461	hypothetical protein	CD305_02491	alpha/beta hydrolase family protein
CD305_02420	Type 1 restriction-modification protein subunit S	CD305_02462	hypothetical protein	CD305_02492	Hypothetical protein
CD305_02421	SAM-dependent DNA methyltransferase	CD305_02463	DNA methylase/DNA helicase	CD305_02493	Putative conjugal transfer protein
CD305_02422	Conjugal transfer protein	CD305_02464	hypothetical protein	CD305_02494	Cro/Cl family transcriptional regulator, XRE family transcriptional regulator, replication initiation protein
CD305_02423	Peptidase P60, cell wall hydrolase	CD305_02465	hypothetical protein	CD305_02495	Cell division protein FtsK/SpoIIIE-family protien Tn916-like
CD305_02424	Transposase/major facilitator superfamily	CD305_02466	CnaB collagen binding protein/TonB-dependent receptor - LPXTG	CD305_02496	MBL-fold metallo-hydrolase/beta-lactamase
CD305_02425	ATP/GTP binding protein (CTn3)	CD305_02467	chromosome partitioning protein parB	CD305_02497	Conjugative transposon protein
CD305_02426	conjugal transfer protein, tcpE family protein	CD305_02468	chromosome partitioning protein parA/sporulation initiation inhibition soj_1	CD305_02498	Conjugative transposon protein
CD305_02427	Hypothetical protein			CD305_02499	Putative collagen binding - homologous to CD3392
CD305_02428	Hypothetical protein				
CD305_02429	antirestriction protein ArdA				
CD305_02430	hypothetical protein				
CD305_02431	hypothetical protein				
CD305_02432	hypothetical protein				
CD305_02433	Cro/Cl family transcriptional regulator, XRE family transcriptional regulator, replication initiation protein				
CD305_02434	Cell division protein FtsK/SpoIIIE-family protien Tn916-like				
CD305_02435	Conjugal transfer protein				
CD305_02436	Conjugal transfer protein				
CD305_02437	collagen binding, Cna B domain, LPXTG protein				
CD305_02438	hypothetical protein/DNA binding protein

**Figure 4:**
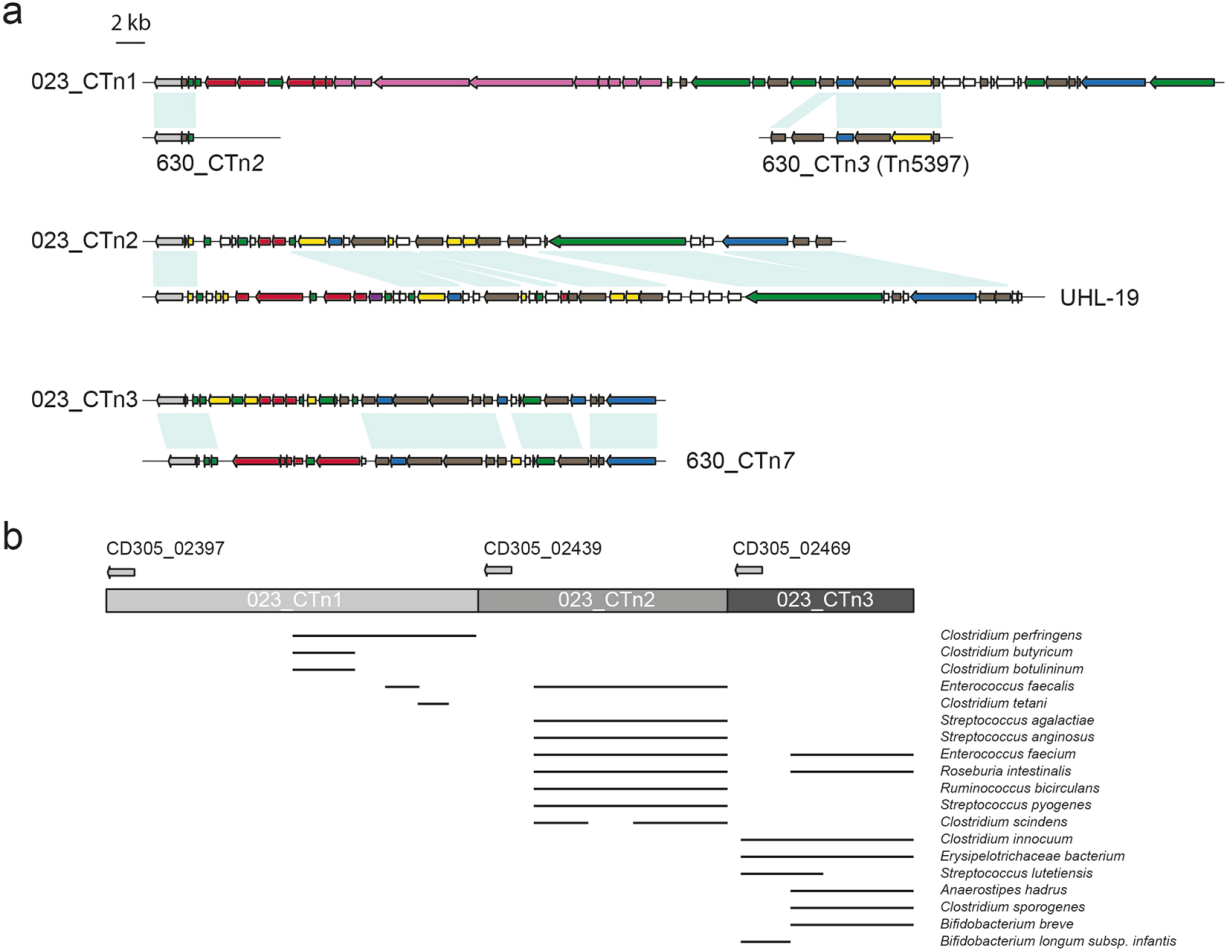
023_CTnT shows sequence identity with *C. difficile* and human microbiome genomes. Regions within 023_CTnT are found within other strains of *C. difficile* and human microbiome genomes. (**a**) Schematic of the three sequential putative transposons within 023_CTnT in CD305; 023_CTn*1*, 023_CTn*2*, 023_CTn*3*. Regions of homology with strain 630 and UHL-19 transposons are indicated below each RT023 transposon. Gene colours indicate putative functions: pale grey, serine recombinase; grey, transposable element/plasmid conjugation; blue, surface proteins and cell wall regulation; green, DNA associated and regulators; red, ABC transporters; purple, signal transduction; pink, biosynthesis/metabolism; yellow, various functional proteins; white, unknown function. **(b**) BLASTn analysis of sequence coverage of 023_CTnT. Each sub-element is represented by a shaded grey box with the serine recombinases shown above indicating the predicted junction between each sub-element. Sequence identities of each species is indicated by a black bar representing >70% sequence identity.

Three serine recombinases are distributed along this gene cluster (CD305_02395, CD305_02439, CD305_02469) (Table 1), which provides evidence that 023_CTnT is potentially comprised of at least three smaller sequential transposable elements, hereafter referred to as 023_CTn*1*, 023_CTn*2* and 023_CTn*3* (Fig. 4a). 023_CTn*1* gene CD305_02397 has low sequence identity to the serine recombinase of 630_CTn*2* and genes CD305_02422–02426 show significant identity to open reading frames 13–17 of 630_CTn*3* (Tn5397) containing conjugation machinery (Fig. 4a). The cargo genes are unique to clade 3 strains and encode putative proteins with no homology to proteins found in other *C. difficile* strains. 023_CTn*2* shows partial sequence identity to the 49 kb chromosomal genetic region observed in the *C. difficile* RT017 1-UHL cluster^17^ (Fig. 4a). In RT017 1-UHL this cluster is inserted within the genomic locus containing CTn7 in strain 630 and contains the CACATGTG palindrome utilised by this transposon in strain 630. This palindrome is absent in 023_CTn*2* which suggests a difference in transfer of these elements. 1-UHL and 023_CTn*2* have some conserved genes (Fig. 4a) but also show divergence in cargo genes, either from evolution of the elements or a difference in acquisition. 023_CTn*3* shows 60% sequence identity to CTn*7* from *C. difficile* 630, mainly in the genes encoding the conjugation machinery and two cargo genes encoding a cell wall hydrolase and the sortase substrate CD3392 (Fig. 4a). The majority of sequence identity resides in the conjugation machinery, with cargo unique to clade 3 strains.

### Clade 3 transposable elements are prevalent in enteric bacteria with novel genes for anaerobic bacteria

BLASTn analysis of the nucleotide region spanning this entire locus reveals a number of regions showing significant sequence identity (>70% nucleotide sequence identity) to sequenced bacterial genomes (Fig. 4b) including *Clostridial* species, *Roseburia intestinalis*, *Streptococcus agalactiae* (Group B Streptococcus, GBS), *Enterococcus faecalis* and *Bifidobacterium longum subsp. infantis*, all of which are found within the microbiome of the human gastrointestinal tract. ICE generally carry accessory genes which provide an advantage to the receiving organism. BLASTP analysis of genes within this genomic island reveals putative genes of lantibiotic ABC transporters, a sortase, three putative collagen binding sortase substrates, transcriptional regulators and a biosynthetic pathway (Table 1). AntiSMASH analysis revealed the biosynthetic cluster in 023_CTn*1* is closely related to a *Streptococcus equi* cluster producing equibactin, a siderophore for iron acquisition^23^, and a similar cluster within *Clostridium kluyveri*^24^.

### Novel elements within RT023 are able to excise from the genome

**Figure 5:**
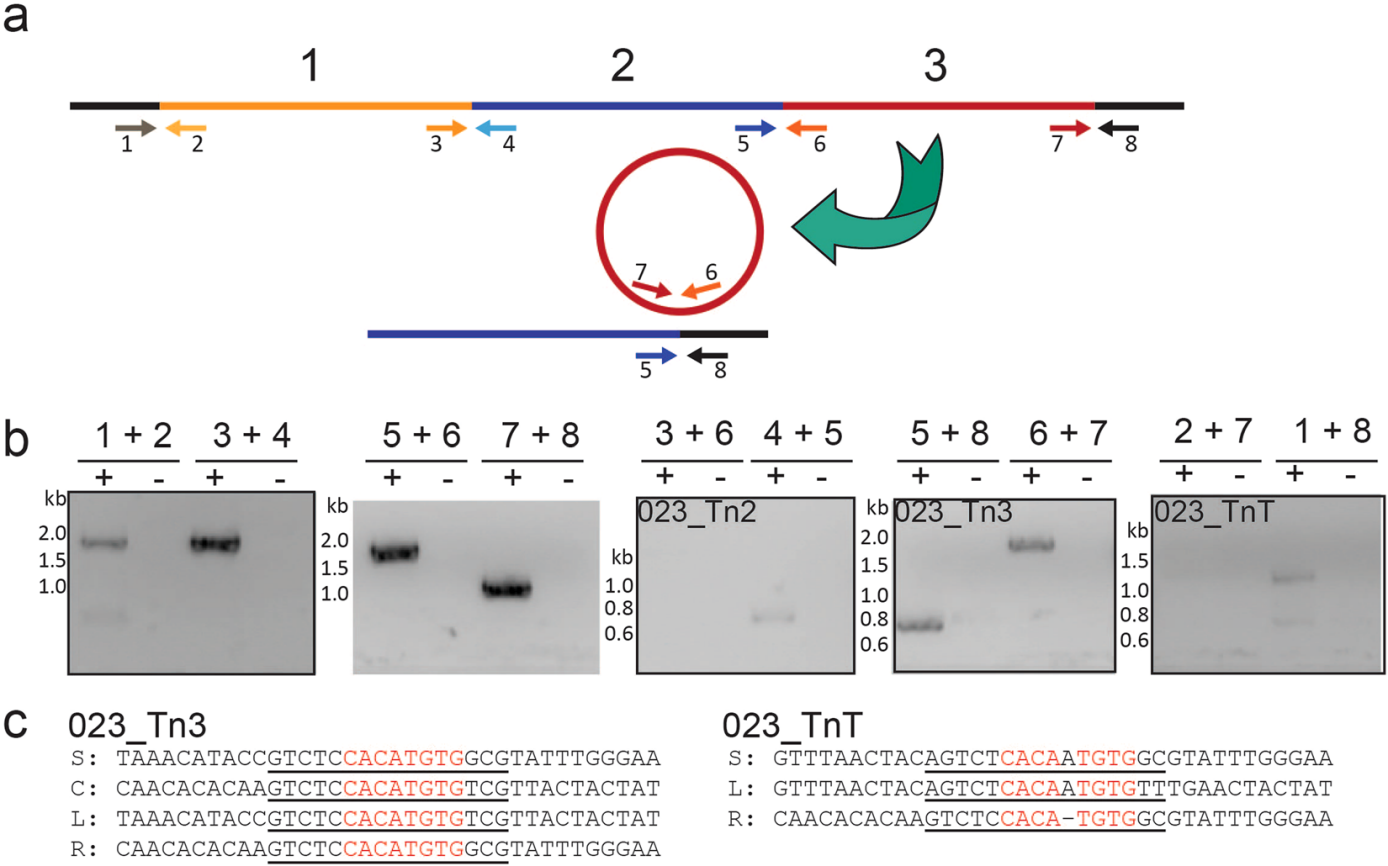
023_CTnT elements are capable of excising. PCR analysis of CD305 genomic DNA confirmed localisation of 023_CTnT and demonstrated some elements are capable of excising from the genome. (**a**) Schematic illustrating primer binding to demonstrate element localisation, empty site and circularised sequences. Junctions could be amplified with 1 + 2, 3 + 4, 5 + 6 and 7 + 8. Empty sites could be amplified with 1 + 4, 3 + 6, 5 + 8 and 1 + 8. Circularisation could be amplified with 2 + 3, 4 + 5, 6 + 7 and 2 + 7. (**b**) PCR analysis of four junctions, empty site and circularisation for 023_CTn*1*, 023_CTn*2*, 023_CTn*3* and the total site (023_CTnT). +, DNA positive; -, DNA negative. (**c**) Sequence analysis of the PCR products for 023_CTn*3* and 023_CTnT. S, site; C, circularisation; L, left junction; R, right junction.

ICE often excise from the genome and form circular structures, which are conjugation and transposition intermediates^15^. Primers were designed to determine if 023_CTnT or any of its constituent parts could circularise (Fig. 5a).

023_CTn*2* was shown by PCR to circularise, however an empty target could not be amplified (Fig. 5b). 023_CTn*3* was clearly shown to circularise and the presence of an empty site shown by PCR (Fig. 5b). Sequencing of the PCR product confirmed that the sequences span from CD305_02469 to CD305_02500 (Fig. 5c). Circularisation occurs across a region flanked by the repeat GTCTCCACATGTGG/TCG covering a palindrome of CACATGTG. Primers flanking the entire region (023_CTnT) also amplify an empty site, which is indicative of mobility of the total region. Excision occurs at a region flanked by CD305_02397 and CD305_02500 at the palindromic sequence CACA(A)TGTG, with the bracketed adenosine only present within CD305_02397 (Fig. 5c). This reflects the empty site observed in outlier strains (Fig. 3) matching the excision system seen for 630_CTn*2*^15^ and is additionally the same 3′ palindrome utilised by 023_CTn*3* within CD305_02500. We were unable to consistently amplify a circular PCR product from the entire region with clear sequencing data spanning each end of the region, suggesting either a low frequency of excision or that stepwise rather than total excision occurs. Therefore, there is evidence that at least 023_CTn*2* and 023_CTn*3* are capable of excising independently, with 023_CTn*3* leaving a clear empty target site.

### 023_CTn3 is able to transfer to other genomes of *C. difficile*

To assess transfer of this region from CD305 to other strains of *C. difficile* ClosTron constructs were designed to target genes within each putative transposon. CD305_02499 within 023_CTn*3* was successfully marked with an erythromycin cassette using ClosTron technology^25^. ClosTrons targeting 023_CTn*1* and 023_CTn*2* were unsuccessful as the ClosTron retargeting plasmids could not be conjugated into a panel of recipient RT023 strains tested despite repeated attempts. Two independent ClosTron mutants of CD305_02499 in 023_CTn*3* of CD305 were chosen for filter mating experiments. To test the ability of 023_CTn*3* to transfer to the non-toxigenic *C. difficile* strain CD37 (Erm^S^, Tc^S^, Rif^R^)^26^ was used as a recipient in filter mating experiments (summarised in Table 2). Erythromycin resistant colonies arose at a frequency of around 10^−7^ transconjugants per donor and recipient (Table 2).

**Table 2:** Frequency of conjugation per donor or recipient (Average of three technical replicates).

Donor	Recipient	Frequency of conjugation/donor With DNase	Frequency of conjugation/donor without DNase	Frequency of conjugation/recipient With DNase	Frequency of conjugation/recipient Without DNase
CD305 (clone 1)	CD37	1.42 × 10^−7^	4.53 × 10^−7^	5.4 × 10^−7^	6.6 × 10^−7^
CD305 (clone 2)	CD37	5.6 × 10^−7^	2.4 × 10^−7^	6 × 10^−7^	2.8 × 10^−7^

Six transconjugants (from three independent filter mating experiments for each ClosTron mutant) were analysed by whole genome sequencing (WGS). This showed that 023_CTn*3* consistently inserts into the CD37 genome at the same location. Insertion occurred within the 630_CTn*7* locus, which in CD37 harbours a transposon with homology to 630_CTn*2* that is lost upon acquisition of 023_CTn*3*. This suggests that transposons can be usurped with selective pressure from the incoming element. The CACATGTG palindrome observed in 630_CTn*7* and 023_CTn*3* is utilised, confirming the method of transfer of this genetic locus. Neither of the two genetic elements proximal to 023_CTn*3* transferred in these experiments.

### Genes within a novel genetic island are expressed in RT023

**Figure 6:**
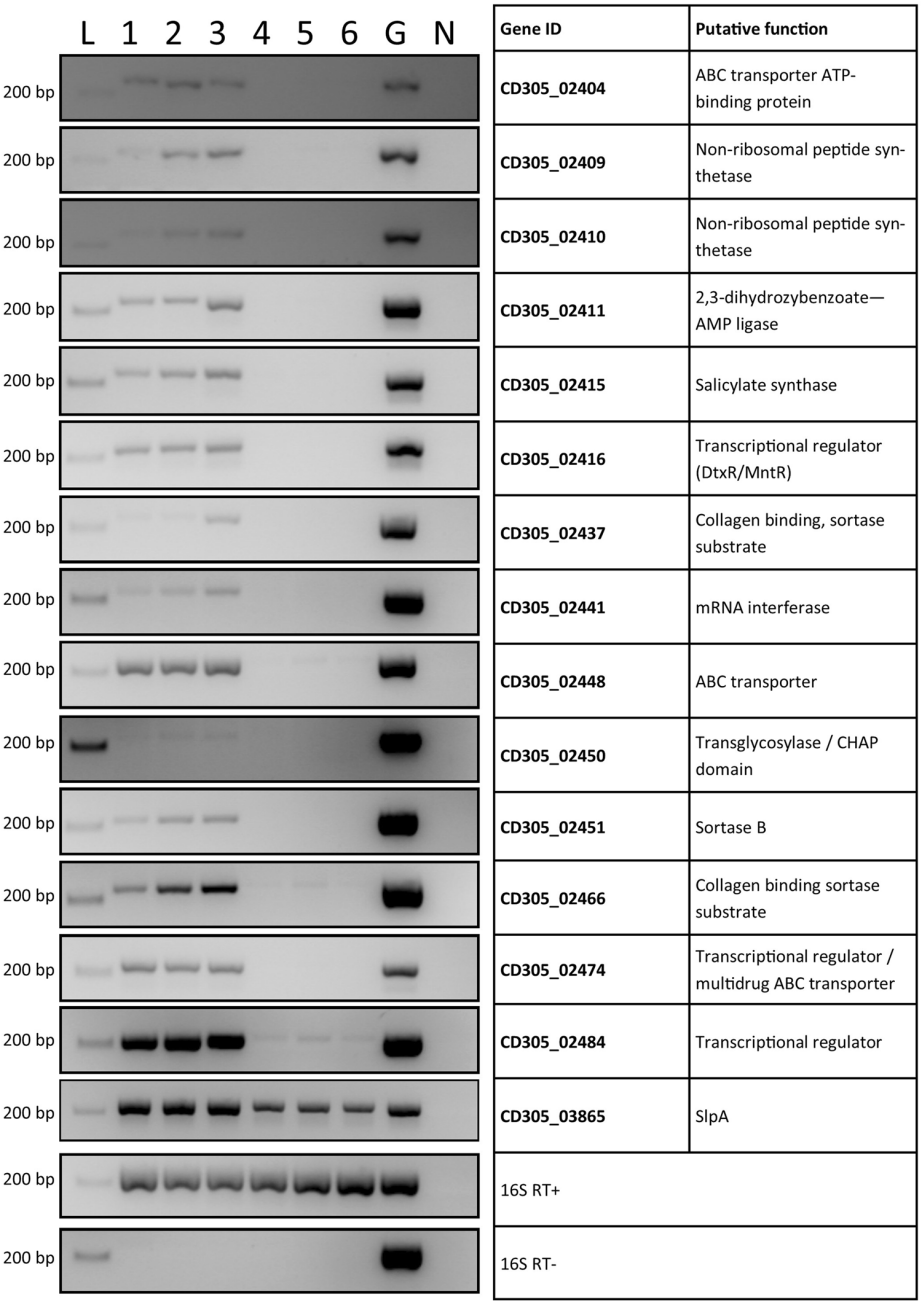
Genes within 023_CTnT are expressed in clade 3 strains. RNA extracted from exponential and stationary phase cultures of clade 3 strains CD305, CZ0502 and SLH89 show expression of fourteen genes within 023_CTnT. 16S PCRs were undertaken on RT+ and RT- samples to show uniform production of cDNA and an absence of gDNA respectively. L, ladder; 1, 2, 3 – exponential cultures; 4, 5, 6 – stationary phase cultures; 1, 4 – CD305; 2, 5 – CZ0502; 3, 6 – SLH89; G, genomic DNA from CD305; N, water negative control. CD305 gene ID and putative functions as indicated.

RNA was extracted from three representative RT023 strains CD305, CZ0502 and SLH89 (from a UK patient, a European patient and a pig isolate respectively) at exponential and stationary growth phases to determine whether genes within 023_CTnT were expressed under laboratory conditions. cDNA was synthesised and 16S PCR on RT+ samples shows uniform production of cDNA across RNA preparations and on RT− samples shows a lack of residual genomic DNA (Fig. 6). 14 genes were selected from within 023_CTnT to determine whether expression was occurring under laboratory conditions, including genes involved in a biosynthetic pathway, its putative transcriptional regulator, an ABC transporter, a sortase enzyme, two putative sortase substrates, and putative DNA binding proteins. Figure 6 shows that most of the genes were expressed well during exponential growth, with CD305_02410, CD305_02437 and CD305_02450 expressed weakly. Meanwhile, most gene expression was diminished or absent by stationary phase, except for the low expression of CD305_02466 and CD305_02484, suggesting evidence of regulation in 023_CTnT as the constitutively expressed core gene *slpA* was expressed well at both growth phases. There were minor differences in expression levels between the three strains but no marked differences except for the two non-ribosomal peptide synthetases (CD305_02409, CD305_02410), which show very low expression in CD305 compared with the two other strains, and putative sortase substrate CD305_02437 which shows higher expression in SLH89. There are no SNPs upstream of these genes to suggest an alteration in transcription profile between strains.

## Discussion

*C. difficile* is a highly diverse species, divided into at least five distinct clades. Clade 3, predominantly made up of RT023 strains, has been less well characterised than the other clades, despite being a prevalent and an important type in Europe causing clinical symptoms similar to hypervirulent strains from RT027 and RT078 and significant recurrence of disease presentation. We conducted WGS analysis on clade 3 strains from our collection and from the literature, which has revealed conserved genetic characteristics that alter the surface architecture of clade 3 and may impact its virulence. Incorporation of a glycosylation cassette into the S-layer locus has been shown previously, which results in deletion of the *cwp2* gene^12^, and removal of *cwp66* promoters^27,28^. This could potentially alter colonisation, however, this may be counterbalanced by the permanent association of core genes collagen binding sortase substrates CD2831 and CD3246 on the surface of clade 3, which may prevent the hypothesised lifestyle switching through the action of PPEP-1 in these strains^7,18^. The incorporation of a second sortase enzyme and three sortase substrates in the transposon region may also enhance the colonisation of these strains. Meanwhile the loss of phage protection provided by the phase-variable surface protein CwpV may result in an increased ability of clade 3 *C. difficile* strains to incorporate foreign DNA into its genome via transduction^5^.

A large genetic island is present within the genome of clade 3 strains. We have demonstrated here that genes within the element are expressed and therefore likely to be utilised *in vivo*, with at least one of the predicted elements able to excise and transfer to another strain of *C. difficile*. Genes along the entire region are prevalent amongst bacteria of the human gastrointestinal microbiome, including *Roseburia intestinalis, Enterococcus faecalis* and *Bifidobacteria* species. *Bifidobacteria* are commonly associated with early colonisation of breast-fed infants^29^. It has been frequently reported that *C. difficile* is a common coloniser of infants without displaying signs of disease, potentially due to the presence of *Bifidobacterium longum*^30^. It is possible that clade 3 strains acquired transposable elements such as these during colonisation of infants and acquisition of these genes could enhance long term colonisation leading to recurrent infections.

The genetic island contains genes for a sortase enzyme and two proximal putative substrates as cargo. There is also a third sortase substrate in 023_CTn*3*, equivalent to the sortase substrate found in 630_CTn*7* (CD3392)^16^. Sortases are enzymes which covalently anchor specific protein substrates to the peptidoglycan cell wall or polymerise pili^31,32^ and are often involved in colonisation and virulence^33,34^. These three sortase substrates are predicted to be collagen-binding proteins and therefore likely to be important in colonisation of the intestine. Sortase substrates as cargo on conjugative transposons is common in *C. difficile*^16^, and CD3392 has been shown to be a substrate of the core genome sortase enzyme^35^. This core sortase has been shown to have specificity for the S/PPKTG motif in substrates, but the two additional sortase genes found in these elements encode for I/TPKTG motifs and therefore may not be substrates suitable for this core sortase enzyme. It is possible that they are substrates for the sortase seen within 023_CTn*2*. Until now it is uncommon for sortase enzymes to be found on conjugative transposons of *C. difficile*, with the only previous evidence in the related element in RT017 strains from a London hospital^17^. The addition of a second sortase is rare in *C. difficile*, with the only known duplicate sortase in the core genome to be within strain 630 (Clade 1). This gene, CD3146, contains a stop codon and is assumed to be a pseudogene. Further study of clade 3 should reveal whether these gene acquisitions enhance colonisation by these strains.

Genes relating to the non-ribosomal synthesis of peptides are found within 023_CTn*1*, which are predicted to synthesise a siderophore, a rare occurrence in anaerobic bacteria. This is likely to be similar in structure to iron binding siderophores yersiniabactin, pyochelin and equibactin synthesised by *Yersinia, Pseudomonas* and *Streptococcus*, respectively^23,36,37^. There is high protein sequence identity to a cluster from *C. kluyveri* producing a ferric iron chelator^24^. The *C. kluyveri* cluster is adjacent to integrase genes associated with conjugative transposons and is therefore likely to be an element with the potential to transfer between different species of bacteria. *C. kluyveri* is not a commensal of the human intestine and was first isolated from mud. *C. novyi* however, which contains homologous genes, is found in soil and faeces. This is the first evidence of such a cluster in the major pathogen *C. difficile*, and the additional iron acquisition properties has the potential to enhance virulence. The transcriptional regulator CD305_02316 has been shown to negatively control the related cluster within *Streptococcus equi* by inhibiting transcription of synthetase genes^23^ but there does not seem to be evidence of a similar relationship within this cluster in *C. difficile* as the regulator and regulated genes are expressed at the same growth phases. The regulation and role of this predicted siderophore in *C. difficile* remains to be determined.

023_CTnT encodes peptides which are homologous to other transcriptional regulators, such as AbrB, a global transcriptional regulator in *Bacillus subtilis* that represses the expression of numerous genes at exponential growth phase^38^. Expression of the AbrB homologue in RT023 does not repress exponential phase expression of proximal genes. However, its presence has the potential to affect wider genome expression in clade 3 strains.

We have shown here that 023_CTnT is able to excise from the genome, with evidence of 023_CTn*2* and 023_CTn*3* circularising, an early step in transfer to other genomes. An empty target for 023_CTn*2* could not be amplified by PCR. This could be due to limited replication of excised 023_CTn*2* so that it is present in a higher copy number than its regenerated target and therefore detectable by PCR, whereas the regenerated target is present in too low a concentration to be detected. Using ClosTron technology we were able to mark 023_CTn*3* and demonstrate its transfer to a non-toxigenic strain of *C. difficile* CD37, proving that at least part of this region is a mobile element. Due to the nature of the accessory genes present, including those encoding collagen binding proteins, it is likely that co-infection with other strains of *C. difficile* in the intestine could lead to wider dispersion of these genes, with the potential for improved colonisation. RT017 strains from a London hospital also contain some cargo genes of 023_CTn*2*, though the direction of DNA transfer is unclear, this demonstrates that these recently described elements are readily transferring between strains of *C. difficile*. The demonstration of the ready transfer of transposons to non-toxigenic strains has implications in the use and safety of non-toxigenic strains as potential live attenuated vaccines to prevent *C. difficile* infection^39^.

This work has shown that RT023 strains of *C. difficile* contain distinctive features on the cell surface including the loss of CwpV and permanence of collagen binding sortase substrates. They also contain a novel genetic element, at least part of which is capable of horizontal gene transfer. This element contains genes which are predicted to enable the host organism to thrive in the gut. Furthermore, bioinformatic analysis of other members of the gut microbiota shows that they have high DNA sequence identity to genes in this element, showing that members of this microbiota have access to a vast gene pool. Our work characterised one of the elements that provides this access.

## Materials and Methods

### Bacterial study isolates and growth conditions

*C. difficile* cultures were cultured anaerobically (Don Whitley Scientific, West Yorkshire, United Kingdom) at 37 °C in BHIS broth (BHI broth (Oxoid) supplemented with 0.1% L-cysteine (Sigma), 0.5% tryptose (Bacto), and 1.5% agar for BHIS agar plates (Bacto).

### Bioinformatic analysis

Nextera XT libraries sequenced on a Miseq sequencing system (Illumina, CA, USA) of 86 clade 3 strains of *C. difficile*^14^ were analysed by BLAST to determine gene function and AntiSMASH to determine the putative function of the biosynthetic cluster within the novel transposon cluster^40^.

### Recombinant protein expression

*PPEP-1* was cloned between NcoI and XhoI sites in pET28a to express the protein with a C-terminal 6xHIS tag. Plasmids were expressed in Rosetta *E. coli* in Overnight Instant TB media (Merck) at 37 °C, with uninduced controls grown in LB broth. Cells were lysed by freeze-thaw and suspension in PBS containing 1/10 BugBuster Protein Extraction Reagent (Novagen), 40 μg/ml DNaseI (Sigma), 500 μg/ml lysozyme (Sigma) and incubated 45 min RT with gentle agitation. Following lysis, the suspension was centrifuged at 14,000 × *g* for 10 min 4 °C. Supernatants containing soluble protein were separated from the insoluble pellet which was solubilised in PBS with 1% SDS and boiled at 100 °C 10 min.

### Cell fractionation

For extraction of peptidoglycan anchored proteins, cultures were harvested at 4,000 × *g* for 2 min, resuspended in PS buffer (sodium phosphate pH 7.0, 0.5 M sucrose) to OD 50/ml with 30 μg/ml endolysin^41^, and incubated anaerobically at 37 °C for 2 hours. Protoplasts were harvested at 6,000 × *g* 20 min RT, the supernatant containing cell wall proteins were transferred to a fresh tube. Culture supernatants were concentrated with 10% TCA 30 min on ice and washed twice with 90% acetone for 15 min with shaking before resuspension in PBS to OD 50/ml. Whole cell lysates were prepared by freeze-thaw at −20 °C of culture pellets, suspended to OD 50/ml in PBS, 40 μg/ml DNaseI (Sigma) and incubated at 37 °C for 1 hour.

### Immunoblotting

Preparations were run on 12% Novex NuPAGE Bis-Tris SDS-PAGE gels (Life Technologies) before being transferred to Hybond-C Extra nitrocellulose membrane (GE Healthcare). Membranes were probed with mouse antiserum against 6xHisTag (1:5000, Abcam), or mouse antiserum against CD2831^20^, followed by goat anti-mouse IRDye conjugated secondary antibody (1:2000, LI-COR Biotechnology). Blots were visualised with an Odyssey near-infrared imager (LI-COR Biotechnology).

### Transposon mobility analysis

Genomic DNA (gDNA) was extracted from 5 ml overnight cultures of strain CD305 grown in BHIS broth from a single colony. Cells were harvested at 4000 × *g* 10 min, resuspended in 200 μl 0.2 M glycine pH 2.2 and incubated at room temperature 20 min with rotation to remove surface proteins and polysaccharides. Cells were harvested at 17,000 × *g* 10 min and the supernatant discarded. Cell pellets were resuspended in 200 μl nuclease free H_2_O with 1.5 mg/ml RNaseA, transferred to 0.1 mm zirconian beads, and lysed with 1 ml CLS-TC (MP Bio) by Ribolyser for 40 s. Suspensions were incubated at 37 °C for 1 hour and then processed with FastDNA Spin kit (MP Bio) and DNA eluted in 100 μl ultra-pure H_2_O. Purified gDNA from three independent extractions were analysed by PCR using the high fidelity Phusion polymerase (NEB). PCR products were sequenced by Sanger sequencing (Source Bioscience).

### Mating experiments

This method is based on filter-matings described by Mullany *et al*.^42^. Cultures of both donor (CD305; independent ClosTron mutants clone 1 and clone 2; Rif^s^, Erm^r^) and recipient (CD37; Rif^r^, Erm^s^) strain were grown for 16 h in pre-reduced BHI broth. These were used to start a 10 ml culture of the donor strain and a 50 ml culture of the recipient, both at an OD600 = 0.1. These were grown shaking at 50 rpm anaerobically. After 4–6 h, when the OD600 was between 0.6 and 0.8, the cultures were centrifuged for 10 min at 4,500 × *g* and the pellets re-suspended in 500 μl pre-reduced BHI broth. The two cultures were mixed, DNase (50 µg/ml) added and 200 µl was spread onto each of four 0.45 µm pore size cellulose nitrate filters (Sartorius, Epsom, UK), on antibiotic free BHI agar. After 24 h the filters were placed into 25 ml tubes and 1 ml BHI broth was added. The tubes were vortexed and the resulting cell suspension was spread onto selective plates containing Rifampicin 25 µg/ml and Erythromycin 10 µg/ml. After 72–96 h the putative transconjugants were counted and sub-cultured onto fresh selective plates. In order to distinguish transconjugants from spontaneous rifampicin resistant mutants we determined if the PaLoc was present or not. As the donor CD305 contains the PaLoc and the recipient CD37 does not (the PaLoc is replaced by a 115 bp non-coding region in this strain) we used PCR to determine if the putative transconjugants contained the 115 bp region (if the PaLoc was present no amplification would be expected as the PaLoc is over 20 kb, too large to be amplified under these conditions) (Braun *et al*., 1996). As the PaLoc is capable of low frequency transfer between bacterial strains (ref) we also amplified PCR products with 400 bp of the *stpK* gene from CD37 transconjugants and CD305 and Sanger sequenced. There are SNPs and indels that differ in this gene between CD37 and CD305 allowing spontaneous mutants of the donor to be distinguished from genuine transconjugants.

### RNA extraction

10 ml cultures of exponential and stationary phase *C. difficile* were incubated with pre-equilibrated RNA protect for 5 min anaerobically and harvested at 4 °C for freezing pellets at −80 °C. Pellets were resuspended in 2 ml RNA pro solution (MPBio), transferred to lysing matrix tubes and processed in the FastPrep Ribolyzer at for 40 s. Samples were centrifuged at 13,000 × g 10 min 4 °C and supernatant transferred to a fresh 2 ml tube. The supernatant was washed once with chloroform and the aqueous phase transferred to an equal volume of 100% EtOH for precipitation overnight at −20 °C. Nucleic acids were harvested at 13,000 × g 4 °C 30 min and washed with 500 μl 70% EtOH before air drying the pellet. RNA samples were treated twice with Turbo DNaseI for 1 h at 37 °C in the presence of RNase inhibitor. Following DNase treatment samples were cleaned with equal volume acid phenol and chloroform washes before precipitation in 3 volumes 100% EtOH overnight at −20 °C. RNA pellets were washed with 300 μl 70% EtOH and air dried before resuspension in 20 μl nuclease free water. Samples were tested for DNA contamination and RNA quality by PCR, nanodrop and bioanalyser. cDNA was produced from with Superscript II with 1 μg of RNA. PCRs were conducted with primers indicated in Supplementary Table S2.

## Supplementary information

Supplementary Information

Supplementary Table S1

Supplementary Table S2

## Data Availability

Sequence data that supports the findings of this study have been deposited in EMBL Nucleotide Sequence Database (ENA) with the Accession Codes ERS2502454 (CD305 reference genome) and study Accession Number PRJEB26893.
