# Supplementary material for: Clostridium difficile clade 3 (RT023) have a modified cell surface and contain a large transposable island with novel cargo
Source: Sci Rep. 2019 Oct 25;9:15330. doi: 10.1038/s41598-019-51628-5 (PMC6814731; doi:10.1038/s41598-019-51628-5)
Supplement: Supplementary file 1 — Supplementary Information [file 41598_2019_51628_MOESM1_ESM.docx]

*Clostridium difficile* clade 3 (RT023) have a modified cell surface and contain a large transposable island with novel cargo

Helen Alexandra Shaw, Ladan Khodadoost, Mark D. Preston, Jeroen Corver, Peter Mullany & Brendan W. Wren

Supplementary Information:

Supplementary Figures S1 – S3: uncropped western-blot images and DNA gels

Supplementary Table S1 – additional excel file

Supplementary Table S2 – additional excel file

Figure 1C


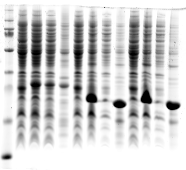

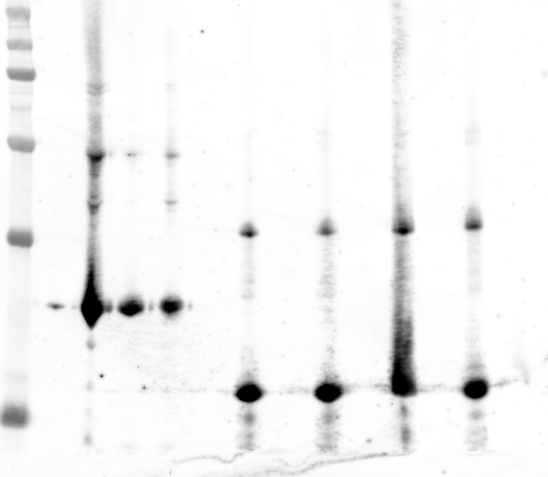


Figure 1D


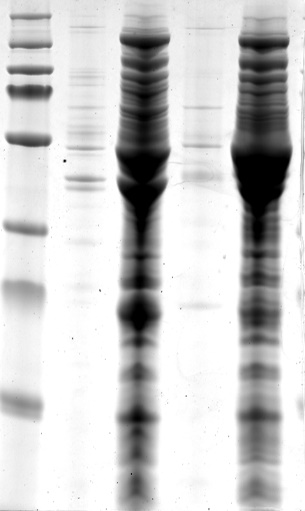

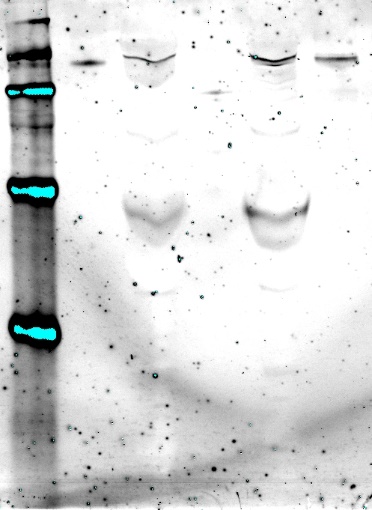


**Supplementary Figure S1:** Uncropped Coomassie gel and Western blot images corresponding to Figures 1C and 1D


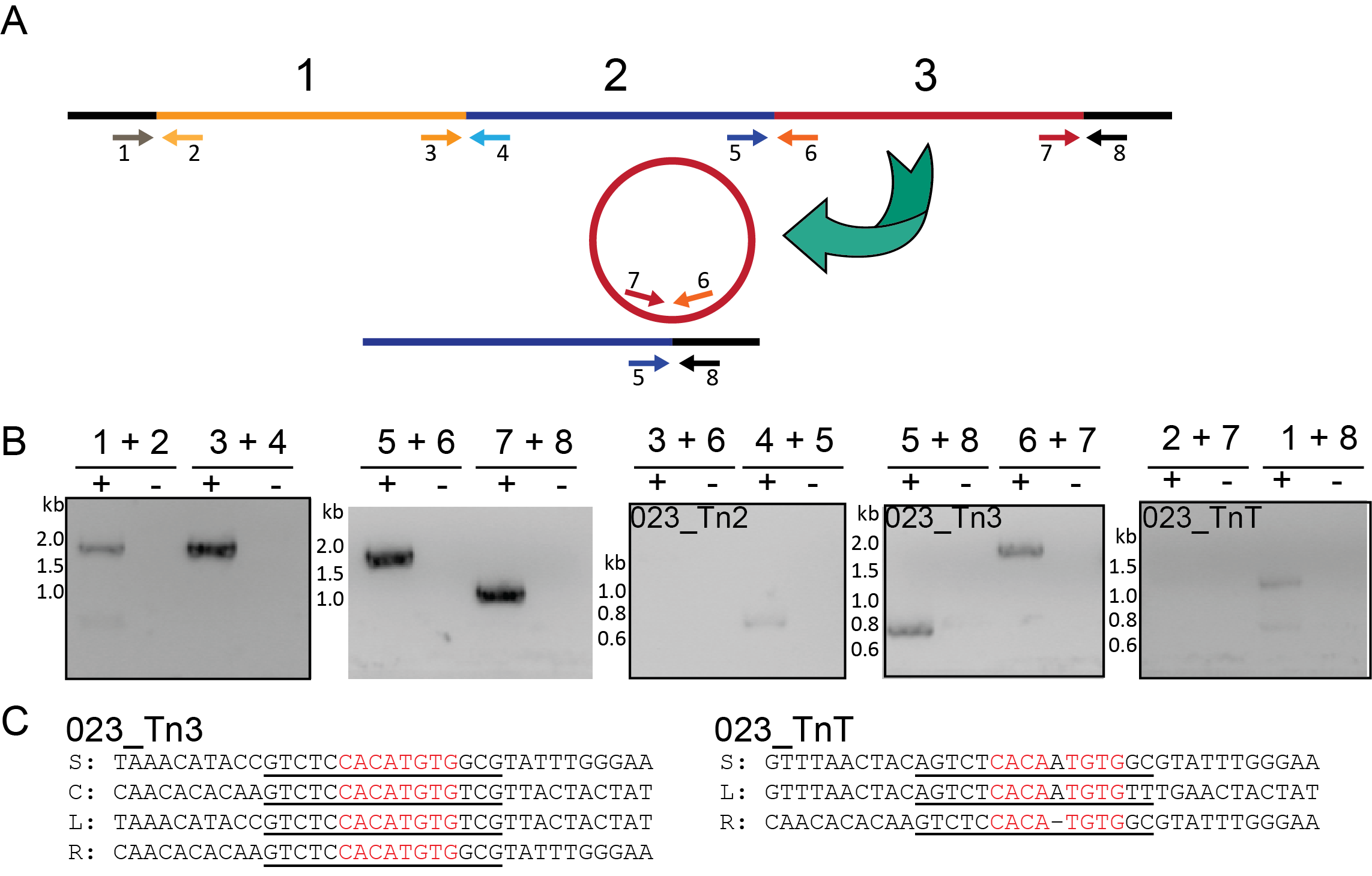

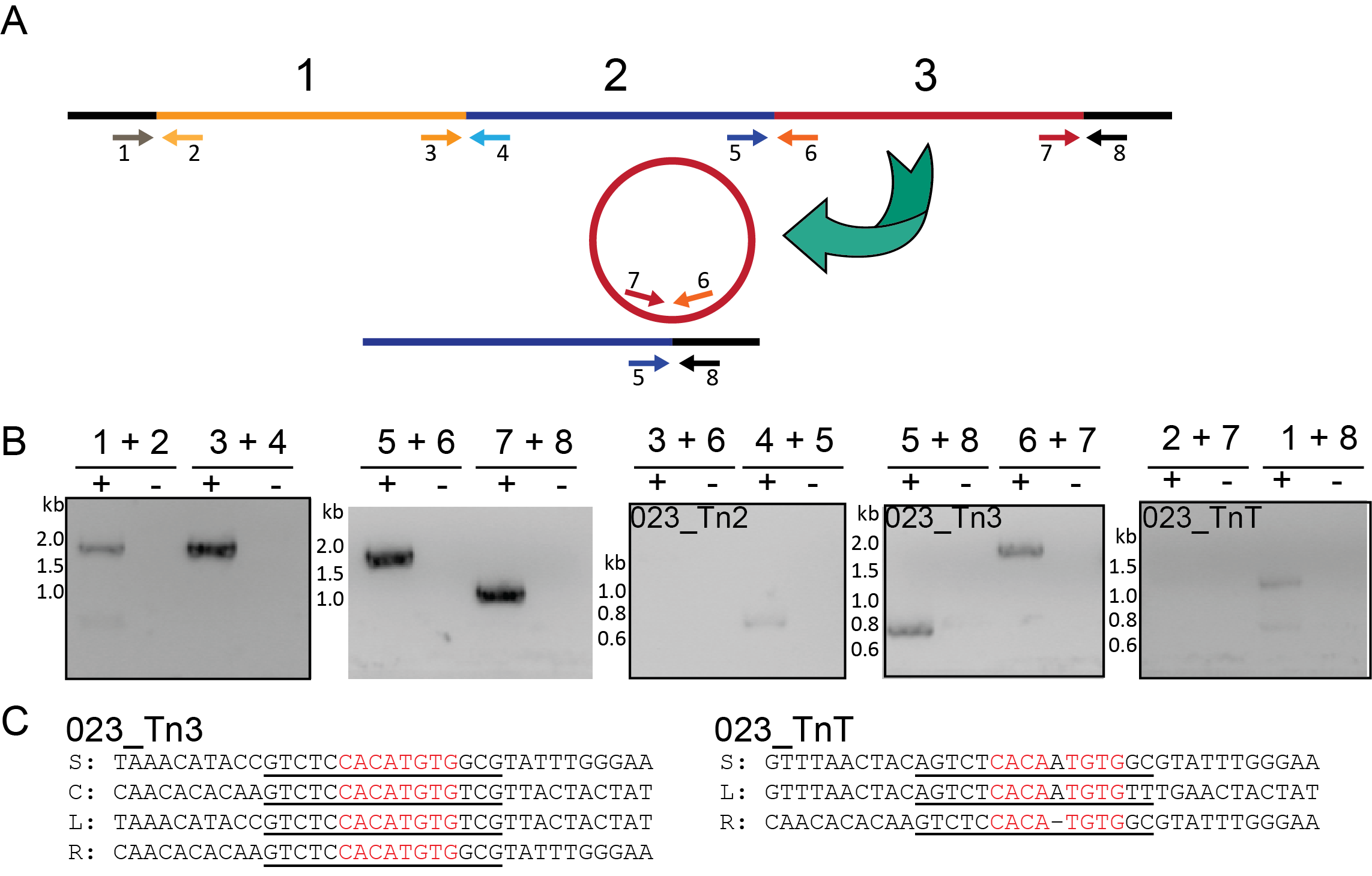

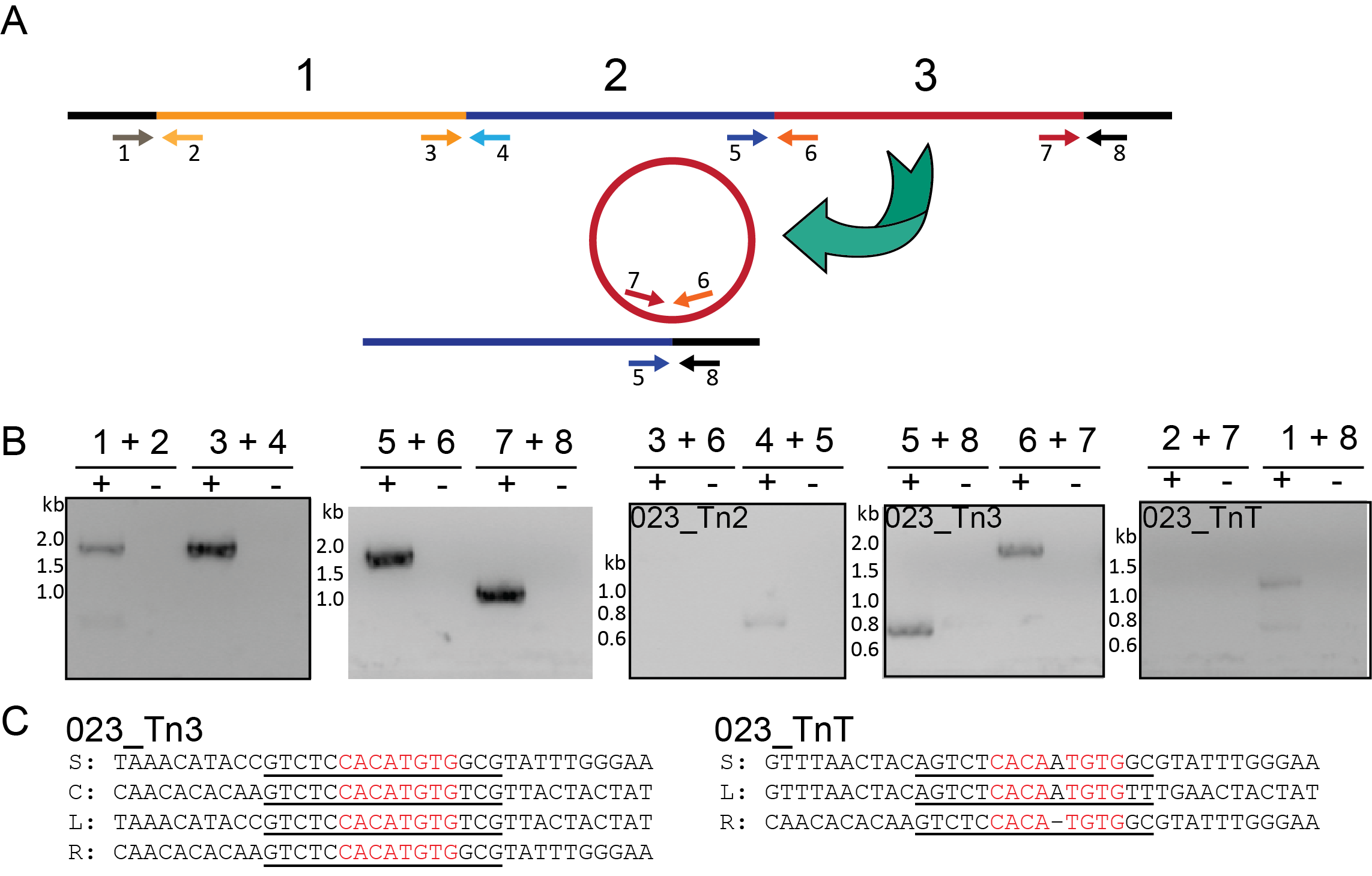

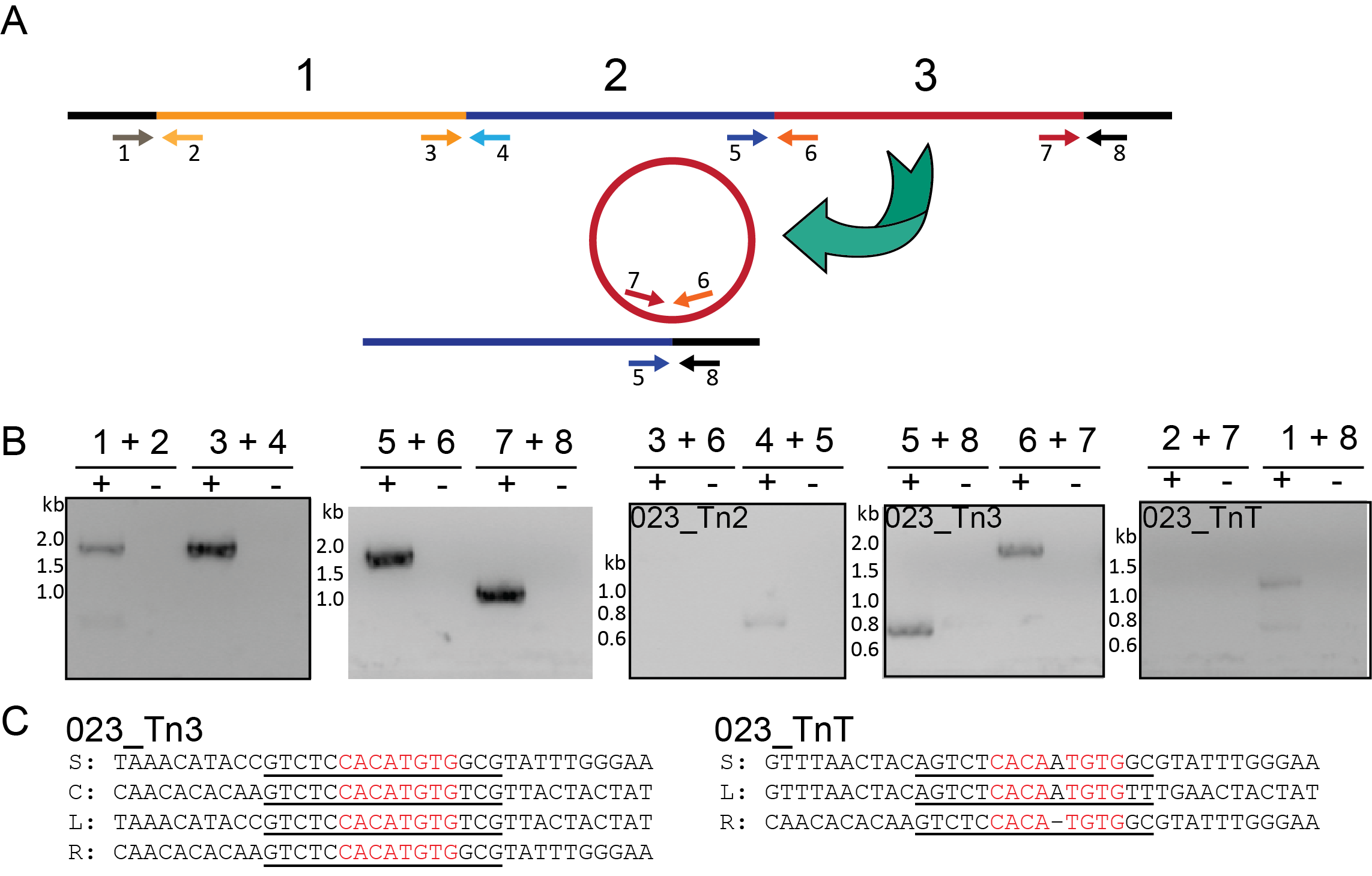
Figure 5B


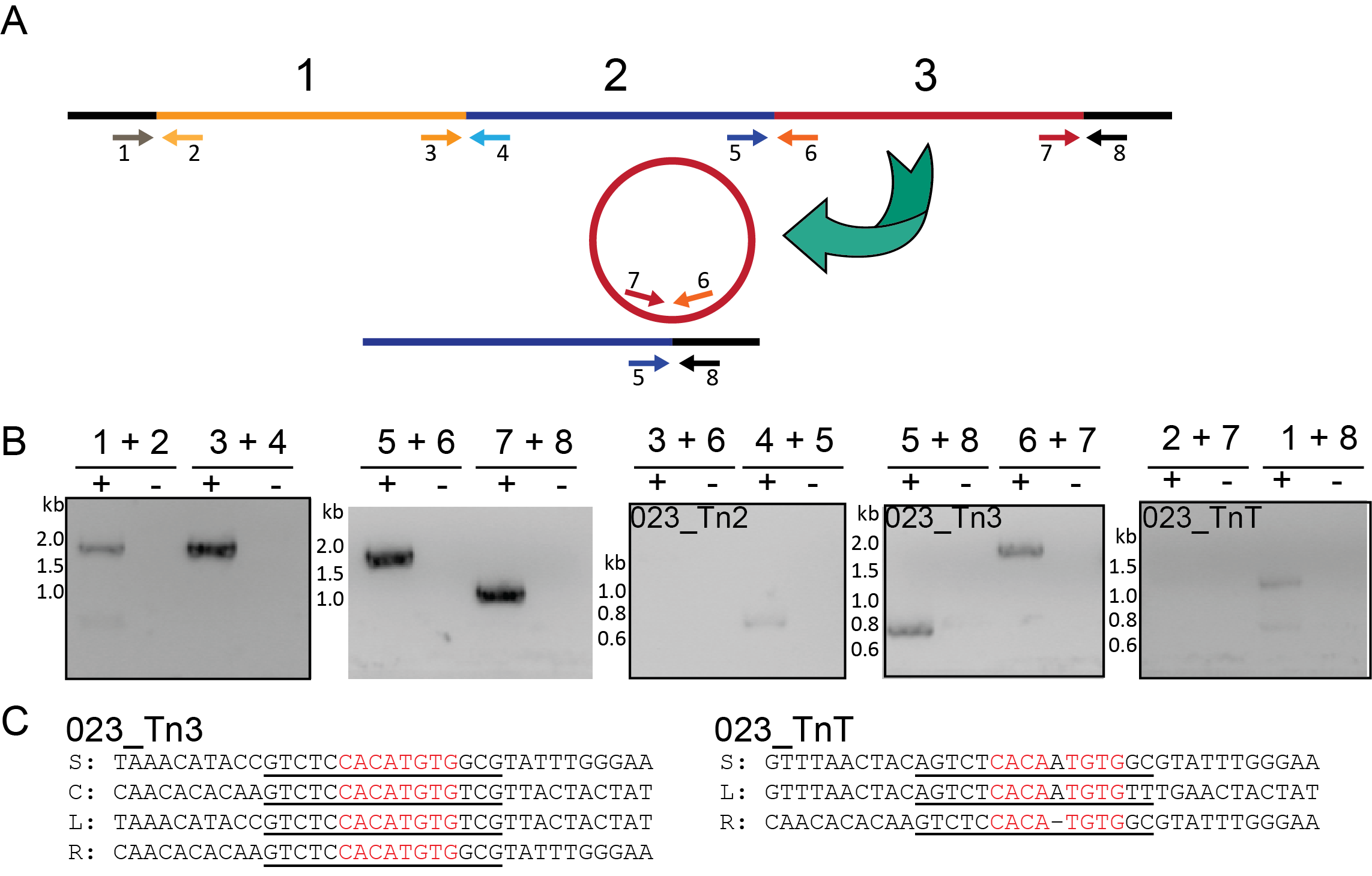

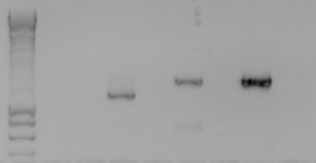

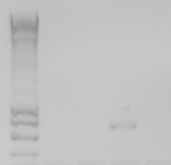

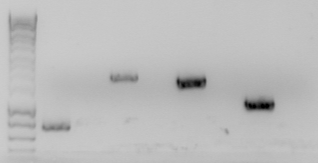

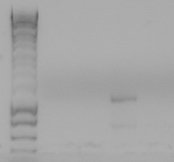


**Supplementary Figure S2:** Uncropped DNA gels of transposon circularisation analysis

Figure 6

16S RT-


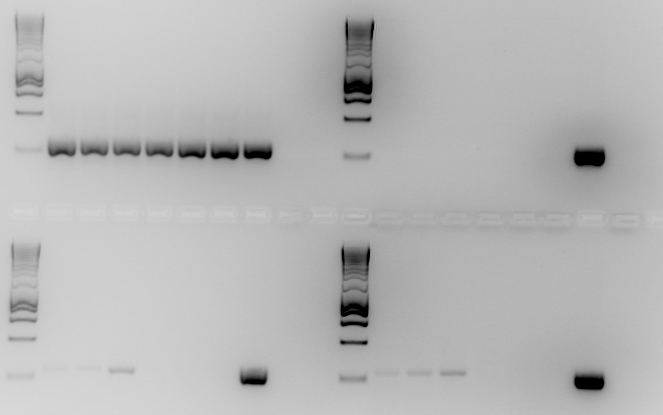


CD305_02441

CD305_02437

16S RT+


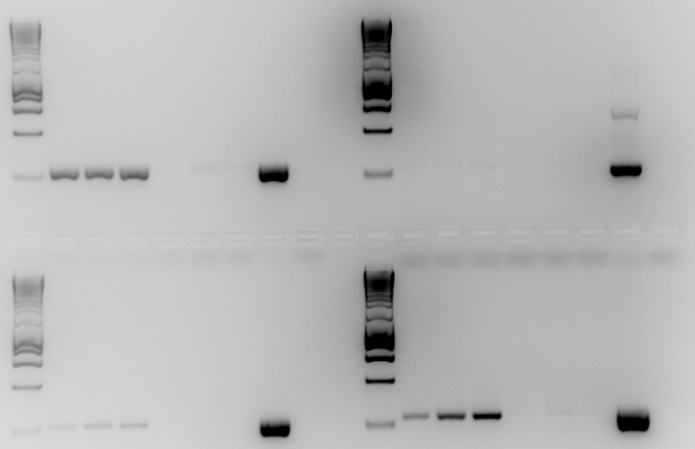

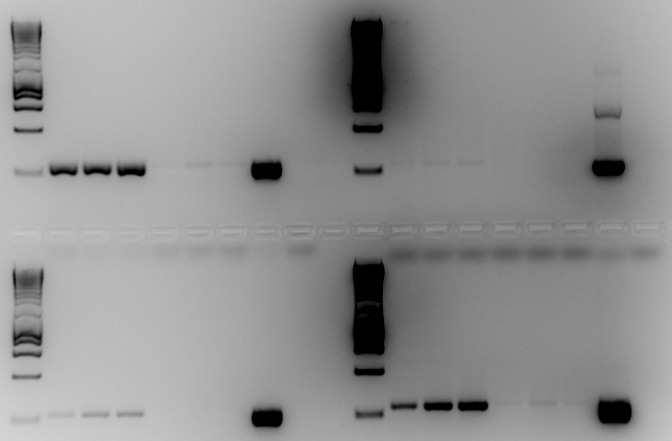


CD305_02466

CD305_02451

CD305_02450

CD305_02448

CD305_02450

CD305_02466

CD305_02451

CD305_02448

Lower intensity image Higher intensity image


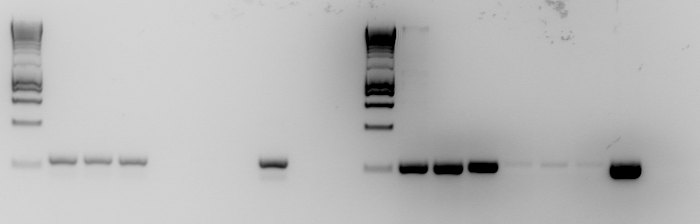


CD305_02474

CD305_02484


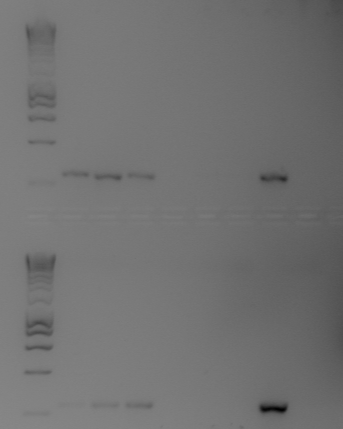

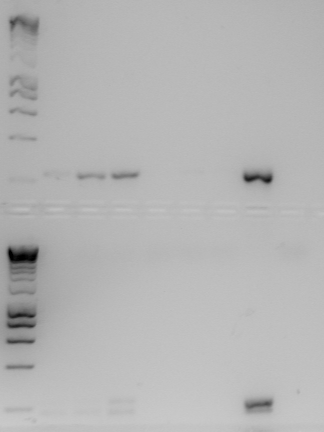


CD305_02411

CD305_02409

CD305_02410

CD305_02404


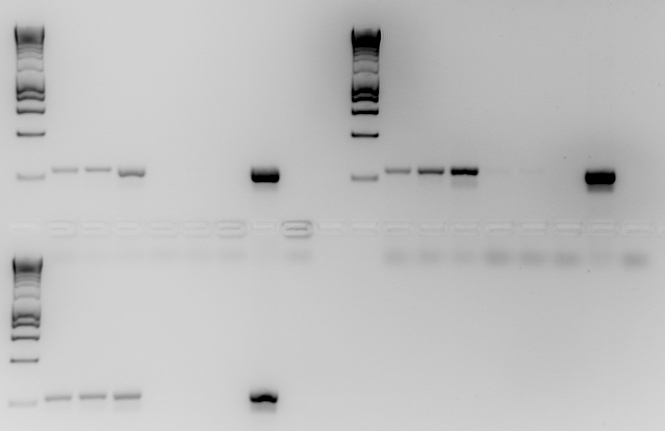


CD305_02416

CD305_02415

CD305_02411

**Supplementary Figure S3:** Uncropped DNA gels from RT-PCR for expression of genes in 023_CTn
